# ELSI practices in genomic research in East Asia: implications for research collaboration and public participation

**DOI:** 10.1186/gm556

**Published:** 2014-05-30

**Authors:** Go Yoshizawa, Calvin Wai-Loon Ho, Wei Zhu, Chingli Hu, Yoni Syukriani, Ilhak Lee, Hannah Kim, Daniel Fu Chang Tsai, Jusaku Minari, Kazuto Kato

**Affiliations:** 1 Department of Biomedical Ethics and Public Policy, Graduate School of Medicine, Osaka University, 2-2 Yamadaoka, Suita, Osaka 565-0871, Japan; 2 Centre for Biomedical Ethics, Yong Loo Lin School of Medicine, National University of Singapore, 10 Medical Drive, Singapore 117597, Republic of Singapore; 3 The Ethox Centre, Nuffield Department of Population Health, University of Oxford, Old Road Campus, Headington, Oxford OX3 7LF, UK; 4 Department of Social Sciences, Fudan University, 220 Handan Road, Shanghai 200433, China; 5 Office of the President, Shanghai Jiaotong University School of Medicine, 227 Chongqing Road, Shanghai 200025, China; 6 Department of Forensic and Legal Medicine, Faculty of Medicine, Padjadjaran University, Jl. Eijkman 38, Bandung 40161, Indonesia; 7 Center for ELSI Research, Asian Institute for Bioethics and Health Law, Department of Medical Law and Ethics, College of Medicine, Yonsei University, 50, Yonsei-ro, Seodaemun-gu, Seoul 120-749, South Korea; 8 Department of Social Medicine, National Taiwan University College of Medicine, 1 Section 1, Jen-Ai Road, Taipei 100, Taiwan; 9 Institute for Integrated Cell-Material Sciences (iCeMS), Kyoto University, Yoshida Ushinomiya-cho, Sakyo-ku, Kyoto 606-8501, Japan

## Abstract

Common infrastructures and platforms are required for international collaborations in large-scale human genomic research and policy development, such as the Global Alliance for Genomics and Health and the ‘ELSI 2.0’ initiative. Such initiatives may require international harmonization of ethical and regulatory requirements. To enable this, however, a greater understanding of issues and practices that relate to the ethical, legal and social implications (ELSI) of genomic research will be needed for the different countries and global regions involved in such research. Here, we review the ELSI practices and regulations for genomic research in six East Asian countries (China, Indonesia, Japan, Singapore, South Korea and Taiwan), highlighting the main similarities and differences between these countries, and more generally, in relation to Western countries. While there are significant differences in ELSI practices among these East Asian countries, there is a consistent emphasis on advancing genomic science and technology. In addition, considerable emphasis is placed on informed consent for participation in research, whether through the contribution of tissue samples or personal information. However, a higher level of engagement with interested stakeholders and the public will be needed in some countries.

## Introduction

Research on the ethical, legal and social implications (ELSI) of human genetics and genomics was originally developed in the context of the Human Genome Project (HGP) [1,2], and it is now applied in other areas of research, such as nanotechnology research and development [3]. Over the past decade, an increasing number of approaches linking social sciences research on bioethical and medical practices have reflected a wider involvement of social scientists, including ELSI researchers, in developing policies for biomedical research that are more responsive to broader social needs [4-12].

With the rapid advances in genomic science and technology, there is a greater need to develop common infrastructures and platforms for international collaboration and public participation. These are necessary not only to meet scientific needs, but also to sustain public trust and support for scientific research locally and internationally. One such initiative is the Global Alliance for Genomics and Health (Global Alliance) [13], launched in 2013 and now involving more than 170 leading organizations in healthcare, research and disease advocacy all over the world. It aims to ‘create a common framework of harmonized approaches to enable the responsible, voluntary, and secure sharing of genomic and clinical data’ [14]. Another more ELSI-focused initiative is ELSI 2.0, launched in 2012 and aimed at enabling ELSI research to ‘become more coordinated, responsive to societal needs, and better able to apply the research knowledge it generates at the global level’ [15]. One of the major challenges they recognize is the need for international ethics harmonization in the requirements of informed consent and privacy protection. These requirements are underscored by international initiatives, including the International Cancer Genome Consortium (ICGC) [16] and the Public Population Project in Genomics and Society (P3G) [17], although these tend to reflect the ethics practices and policies in North America and Western Europe (hereafter, generally referred to as ‘Western countries’). This is understandable, given the geographical distribution of many of the members and experts involved in these initiatives.

However, ethical, legal and social concerns may differ in other global regions, and it is important to understand these differences in order to facilitate international research collaboration. Here, we aim to review current ELSI practices and regulations relating to human genomic research in a selection of East and Southeast Asian countries, namely China, Indonesia, Japan, South Korea, Singapore and Taiwan (hereafter, generally referred to as ‘East Asian countries’). Our focus on a selection of East Asian countries is largely due to the availability of experts and of policies in the public domain. In addition, a level of demographic distribution is achieved with these East Asian countries, and each of these is actively engaged in genomic research and (with the exception of Indonesia) has launched or is about to launch a population biobank [18]. It is envisaged that other countries in the region, including Thailand, Malaysia and the Philippines, which are increasingly engaged in genome research, will subsequently be included in ongoing efforts to develop ELSI practices that facilitate international collaboration.

A recent report on ELSI studies published in 2003 to 2008 showed that the dominant approach could be characterized as ‘downstream’, in the sense that the concerns followed developments in genomic research and practice [19]. Following the dominant approach, we first review ELSI practices and regulations in East Asian countries relating to the main human genomic research centers and resources, laws and regulations for biomedical and genomic research, ELSI practices and challenges for ethics review and informed consent, and for sample and data sharing (and privacy protection). We then highlight the main differences in ELSI practices between these East Asian countries and Western countries, particularly regarding research infrastructure, regulatory frameworks, ethics review and informed consent. Finally, we discuss the progress and challenges for research collaboration and for public participation in national and international genomics projects.

## ELSI practices in East Asia

Here, we summarize current ELSI practices and policies for genomic research in China, Indonesia, Japan, Singapore, South Korea and Taiwan, highlighting specific issues regarding ethics review and consent processes, and data sharing and protection. For each country, the main centers and resources for genomic research are summarized in Table 1, and current regulations and policies are summarized in Table 2.

**Table 1:** East Asian centers and resources for human genomic research

**Country**	**Institutes conducting large-scale genomic research**^**a**^	**National human genome databases**	**National biobanks**^**b**^
China	• Chinese National Human Genome Center, Shanghai (‘South Center’)	• Pan-Asia Population SNP Database [20]	• Human Genetic Resources Platform (2003–2007) [21]
	• Chinese National Human Genome Center, Beijing (‘North Center’)	• 1000 Genomes Project	• Kadoorie Study of Chronic Disease in China (KSCDC; 2004–2008) [22]
	• Beijing Genomics Institute (BGI), Shenzhen		• National Clinical Specimen Biobank (2011–2015) [23]
Indonesia	• Biotechnology Research Centre of the Science Institute of Indonesia (LIPI)	(Individual institution-wide databases only)	
	• Eijkman Institute		
	• Institute of Tropical Disease		
	• Consortium on Vaccine Research (16 universities and research institutions)		
Japan	• University of Tokyo	• Japanese Genotype-phenotype Archive (JGA) [24]	• Biobank Japan (2003-) [25]
	• Yokohama City University	• Japanese Single Nucleotide Polymorphisms (JSNP)	• National Center Biobank Network (NCBN; 2012-) [26]
	• RIKEN Center for Genomic Medicine (CGM)	• Human Genome Variation Database (HGVD)	• Tohoku Medical Megabank (ToMMo; 2012-)
	• Tohoku University	• National Registry of Diseases Office (NRDO)	
	• National Cancer Center		
Singapore	• Genome Institute of Singapore		
	• Bioinformatics Institute		
	• Bioprocessing Technology Institute		
	• National University of Singapore		
	• Nanyang Technological University		
	• National Cancer Centre		
	• Defence Science Office		
South Korea	• Korea Centers for Disease Control and Prevention (KCDC)	• Korean Genomic Variant Database (KGVDB)	• Korea Biobank Project (KBP; 2008-; including Korea Biobank Network and National Biobank of Korea (NBK)) [27,28]
Taiwan	• Genomics Research Center (GRC), Academia Sinica		• Taiwan Biobank (2005-) [29]
	• National Health Research Institute (NHRI)		

^a^A selection of major institutes is included.

^b^Commencement year and termination year are shown in parentheses.

**Table 2:** ELSI practices and regulations for human genomic research in East Asia

	**Regulations**^**a**^	**Responsible agencies**	**Challenges for research collaboration and public participation**
China	Interim Measures for the Administration of Human Genetic Resources (1998) [30]	Ministry of Science and Technology; Ministry of Health	Public concerns about the misuse of genetic information
			Public distrust of authorities
	Regulations on Ethics Review of Biomedical Research Involving Human Subjects (2007) [31]	Ministry of Health	
Indonesia	National Guideline on Research Ethics: Genetic Research (2008)	National Committee on Research Ethics	Protection of rights and privacy of indigenous populations
	National Regulation on Material Transfer Agreement (2009)	Ministry of Health	Need for more integrated approach on genome research
			Low public awareness on genetic information and related ELSI issues
Japan	Ethical Guidelines for Human Genome/Gene Analysis Research (2001)	Ministry of Education, Culture, Sports, Science and Technology; Ministry of Health, Labour and Welfare; Ministry of Economy, Industry and Trade	Public concerns about privacy protection in relation to genetic information
	Protection of Personal Information Act (2003)	Consumer Affairs Agency	
Singapore	Guidelines for IRBs (2004)	Bioethics Advisory Committee/Ministry of Health	Public concerns about potential discrimination, inequitable access and conflicts of interest
	Guidelines on Genetic Testing and Research (2005)	Bioethics Advisory Committee/Ministry of Health	
	National Registries of Disease Act (2007)	National Registry of Diseases Office (NRDO)	
	Guidelines on Use of Personal Information in Biomedical Research (2007)	Bioethics Advisory Committee/Ministry of Health	
	Personal Data Protection Act (2012)	Data Protection Commission	
South Korea	Bioethics and Biosafety Act (2005)	National Bioethics Committee	Low public awareness of genomic medicine
	Personal Information Protection Act (2012)	Ministry of Public Administration and Security	
	Guidelines for Protecting Genomic Information in Medical Institutions (2012)	Ministry of Public Administration and Security; Ministry of Health and Welfare	
	Guidelines for Protecting Genomic Information (2013)	National Project for Personalized Genomic Medicine 21 (PGM21) [32]	
Taiwan	Guidelines for Collection and Use of Human Specimens for Research (2002)	Ministry of Health and Welfare	Insufficient public communication and trust
			Strict legal regulations due to privacy concerns from human right groups
	Regulations on Human Trials (2009)	Ministry of Health and Welfare	
	Personal Information Protection Act (2010)	Ministry of Justice	
	Human Biobank Management Act (2010) [33]	Ministry of Health and Welfare	
	Human Subjects Research Act (2011)	Ministry of Health and Welfare	

^a^Year of establishment of regulation, or enactment of law, is shown in parentheses.

### China

There are three main institutions in China with large genomic databases and biobanks: the Chinese National Human Genome Center in Shanghai (‘South Center’), the Chinese National Human Genome Center in Beijing (‘North Center’), and the Beijing Genomics Institute (BGI) in Shenzhen (Table 1).

Although China has no legislation on human genome research, it has rather strict regulations on research involving human subjects and on the use of human genetic resources (Table 2). Ethics review committees (ERCs) were developed in the 1990s with the launch of international cooperative programs to learn about institutional review board (IRB) systems for human subject research in other countries. The first *Regulations on Ethics Review of Biomedical Research Involving Human Subjects* were issued in 1998. The Ministry of Health (MOH; currently the National Health and Family Planning Commission) issued a revised draft in 2007, and the revision process is still ongoing [31]. According to these regulations, each research institution should set up an ERC when conducting research, and ERC members should be composed of a wide variety of internal and external experts taking into account gender balance. The new *Interim Measures for the Administration of Human Genetic Resources*, originally issued by the Ministry of Science and Technology (MOST) and MOH in 1998, was drafted in October 2012 [30] and is currently the subject of a public consultation.

The ERC system in China is established at three different levels: first within research institutions, then at the municipal and provincial level, and then at the level of the MOH. The ERC at a higher level bears the responsibility for supervising the work of ERCs at lower levels. The regulations described above require institutions and researchers to obtain informed consent from potential donors before collecting and storing samples. The commercialization of genomic research can involve expedited processes that may undermine the individual right of informed consent. In the case of biobanking, the increasing use of general consent or even blanket consent may raise questions about the sufficiency of consent if the possibility of commercialization is not clarified at the outset [34]. This also raises concerns about whether researchers and ERC members are ethically qualified for conducting and reviewing research. These concerns are made worse by a rather unstructured oversight system of local ERCs - each local health bureau organizes a group of experts to conduct an annual evaluation of ERCs, but there is no oversight of the different ERCs.

The National Clinical Specimen Biobank Project [23] has established a biobank network of clinical biological resources in Beijing, Shanghai and other regions. One major biobank is the Kadoorie Study of Chronic Disease in China (KSCDC), which aims to establish a blood-based health database [23]. The other is the Human Genetic Resources Platform established under the leadership of MOST [21]. There is little research collaboration for data access or data sharing, as public distrust of expert authorities has been an obstacle to individual donation of samples to researchers and doctors [35]. The *Interim Measures* define that international collaborative projects involving human genetic resources should apply the principles of mutual benefit and credit, and any consequent patent rights that arise shall be owned by both Chinese and foreign institutions (if any).

Another major challenge for public participation in genomic research is concern over the misuse of genetic information. For example, genetic testing for thalassemia for the recruitment of civil servants in Guangdong province has caused genetic discrimination against people from that province, as they are suspected to be more likely to be thalassemia gene carriers than those living in other areas [36-38].

### Indonesia

Human genome research in Indonesia started to flourish in the 1990s in many leading universities and also in national research institutes, such as the Biotechnology Research Centre of the Science Institute of Indonesia, Eijkman Institute and the Institute of Tropical Disease (Table 1). Subsequently, the number of genomic research protocols has increased substantially. Research funding from the government is usually based on routine medium-scale annual grants, and national large-scale projects such as the National Consortium on Vaccine Research are still uncommon. There are no public biobanks or databases for genomic research in the country yet, and each center manages its own bio-repository and databases.

While Indonesia does not have specific legislation on genetic data, a number of related laws and guidelines on human rights, medical practice and medical education have specific emphasis on group consent targeting human specimen collection from indigenous tribes or traditional communities (Table 2). They also restrict the conduct of genetic screening and prenatal diagnostics on these populations or communities.

The Indonesian research ethics committees are usually institutional (IRBs), affiliated to hospitals, medical schools, or research institutions, and mainly focus on institutional needs. Currently there are 54 IRBs. Larger and more established IRBs accept submission from other institutions. While IRBs are established as independent bodies, they are still highly dependent on their main institutions for funding and staff. The National Research Ethics Committee (KNEPK) was established by the Ministry of Health in 2003. Besides endorsing the establishment of IRBs, KNEPK provides supervision for local IRBs, facilitating continuing education for IRB members, developing research ethics guidelines and facilitating national and international networks on health research ethics. In this sense, it provides consultation for IRBs in special cases. There have been recommendations for KNEPK to become the national supervisory body for IRBs, commencing in 2015. To ensure quality of review, several IRBs have joined the Strategic Initiative for Developing Capacity in Ethical Review (SIDCER) Recognition Programme [39] facilitated by WHO-TDR (World Health Organization, Special Programme for Research and Training in Tropical Diseases) [40] and FERCAP (Forum for Ethical Review Committees in the Asian and Western Pacific Region) [41].

As a consequence of the disagreement between the Indonesian government and WHO regarding H5N1 biospecimen transfer in 2006, the international transfer of biomaterials became a sensitive issue relating to specimen ownership, property rights, benefit sharing and international research collaboration [42,43]. The dispute led to the revision of the *Health Law* (2009) and the enactment of the Ministry of Health Regulation on Material Transfer Agreement (2009). This is intended to ensure fairer benefit sharing, protection of the nation’s sovereignty and strengthening of local researchers’ bargaining position in international research collaborations. The disagreement resulted in a resolution from Indonesia, backed by Malaysia, Thailand and other developing countries to advocate recognition of principles of sovereign right over genetic resources [44]. This led to the promulgation of the *Nagoya Protocol on Access to Genetic Resources and the Fair and Equitable Sharing of Benefits Arising from their Utilization* (the Nagoya Protocol, first adopted in Nagoya, Japan) as a supplement to the Convention on Biological Diversity in 2010. A law was passed to give regulatory effect to the Nagoya Protocol in May 2013. Although the protocol focuses on natural resources in general, it uses the H5N1 issue to endorse strengthening of ‘a fair, transparent, equitable, efficient, and effective system’ for both specimen and benefit sharing. The law demonstrates the country’s focus on protection against biopiracy of genetic resources, including clinical specimens.

Due to great socio-economic disparities within patient populations, IRBs must remain vigilant in ensuring that participation of research subjects is voluntary and on an informed basis. There is also the significant challenge in protecting indigenous populations from exploitation, whether by researchers in Indonesia or overseas. This situation requires public education on international research collaborations and empowerment by a wide range of research and policy experts.

### Japan

Many research institutions in Japan, including the University of Tokyo, Yokohama City University and the RIKEN Center for Genomic Medicine (CGM), conduct personal genome analysis, for example, through whole exome sequencing (Table 1). Genome cohort studies in the Tohoku Medical Megabank Organization (ToMMo) were launched in 2012, and ToMMo has initiated a three-generation cohort study and a community resident cohort study involving 150,000 participants. Whole genome sequencing has already been completed for 1,000 participants at the end of 2013.

Human genome research in Japan is regulated by the *Ethical Guidelines for Human Genome/Gene Analysis Research* (‘*Genome Guidelines*’), which were established in 2001 on the basis of *Fundamental Principles of Research on the Human Genome* (Bioethics Committee, Council for Science and Technology, 2000) issued by the Ministry of Education, Culture, Sports, Science and Technology (MEXT), the Ministry of Health, Labour and Welfare (MHLW) and the Ministry of Economy, Trade and Industry (METI) (Table 2). Such guidelines permit researchers to obtain ‘comprehensive consent’, whereby informed consent is granted not only to a specific and defined project, but also extends to other genome analysis or to other related medical research [45]. The *Protection of Personal Information Act* (2003) is also applicable to the conduct of human genome research, reflecting public awareness of privacy protection [46]. Under this legislation, de-identified genetic information is considered to be personal information as long as the means of re-identification (such as the correspondence table linking samples to original sample donors) is kept in the same institution where the genomic data are handled. In the latest *Genome Guidelines* revised in 2013, ERCs play a more crucial role in several key decisions on the scope of informed consent, the use of existing samples and the return of research results to sample donors. The Medical University Ethics Review Committee Association, administrated in the Life Science and Bioethics Research Center, Tokyo Medical and Dental University, has facilitated practical information exchange between ERC members since 1988.

There are several organizations that manage large-scale biobanks. The most internationally recognized of them is Biobank Japan [25], and its first cohort comprised samples collected from 200,000 patients. The National Center Biobank Network (NCBN) [26], which includes the six national centers, has started to integrate their activities and to accelerate the efficient use of the collected samples. Similar to national databases, the Japanese Genotype-phenotype Archive (JGA) [24] was created in 2013 to share personally identifiable genotype and phenotype data, and this is conducted in partnership with the National Bioscience Database Center (NBDC) and the DNA Data Bank of Japan (DDBJ).

There are at least three challenges to the progress of human genome research: recruitment of healthy individuals, policymaking for returning results, and links between genetics databases and electronic health records (EHRs). In Japan, much of the early genomic research involved associational analysis that linked human genome sequences with particular diseases. In recent years, the Japanese government has, in collaboration with Biobank Japan, NCBN and ToMMo, increasingly invested in genome cohort studies and biobanks for the development of a comprehensive database for populations, including healthy individuals, by integrating health and lifestyle data with data from genomic and proteomic analysis. To facilitate such research, the government continues discussion on the introduction of a law relating to the utilization and protection of EHRs under on-goingreforms on tax and social security. Another important consideration is that public trust and engagement generally need to be improved, especially following the catastrophic earthquake and tsunami in 2011.

### Singapore

A number of research institutes operating under the Agency for Science, Technology and Research (A*STAR) conduct a range of human genomic research in Singapore, including the Genome Institute of Singapore, the Bioinformatics Institute, and the Bioprocessing Technology Institute (Table 1). There are also universities, medical research institutions and major research hospitals undertaking different types of genomic research. An initial attempt to establish a national biobank in 2011 was not successful because of underutilization and the financial burden of maintenance [47]. Instead, major healthcare and research institutions continue to be the key repositories of biomaterials, and these are accessible to researchers (subject to certain requirements, including IRB approval). Although not an exclusively genomic database, the National Registry of Diseases Office is a repository of data that has been used for genetic research.

The Bioethics Advisory Committee (BAC) is a high-level expert body that advises the government on ethical, legal and social issues that arise from biomedical research. It was established in 2000 as a policy measure to safeguard the reputation of scientific work and medical services in Singapore. Since then, the BAC and government agencies (especially the Ministry of Health) have established a general ethical and regulatory framework to ensure appropriate oversight whilst avoiding the over-regulation of research. The *National Registries of Disease Act* was enacted in 2007, to a large degree attributable to a recommendation of the BAC to provide a firm legal footing for disease registries that use personal information in public health research. This recommendation was published in a report by the BAC on the use of personal information (which includes genetic information) in biomedical research [48]. The *Personal Data Protection Act* was enacted in 2012. The legislative provisions are broadly similar to those of the *Data Protection Act* of the UK, and informed consent is emphasized for the use of personal information in research [49].

Within this ethico-legal framework, all human genetic research requires the approval of an appropriately constituted IRB. Participation in research must be on a voluntary and informed basis, and consent is required from the person from whom biological material was obtained or to whom identifiable information (including genetic information) relates [50]. As a general requirement, identifiable information that is used for research should be de-identified as far and as early as possible, and should be stored or transferred as de-identified information. However, personal information that has been irreversibly de-identified need not be subject to privacy and confidentiality requirements. Where applicable, privacy and confidentiality safeguards should be commensurate with the potential risk of harm from disclosure, and should be proportional to the sensitivity of the information and the kind of research being carried out [48,51]. The general framework serves to set out baseline standards for IRB members and researchers, but there are institutional variations as academic, healthcare and research institutions are free to adopt more stringent ELSI requirements and practices.

At the national level, concerns over conflicts of interest have been raised due to a growing emphasis on industrial collaboration and commercialization of research. These could even discourage participation in research. In addition, there is no anti-discrimination legislation in Singapore, and this could also discourage some individuals from undergoing genetic testing, whether for research or for medical purposes. There are also concerns with the application of whole genome and exome sequencing for research and medical purposes. The BAC and Singapore’s Ministry of Health are in the process of updating existing guidelines on genetic testing, genetic research and biobanking.

### South Korea

The Korea Centers for Disease Control and Prevention (KCDC), a government agency belonging to the Ministry of Health and Welfare (MoHW), is the leading institution on the use, management and storage of human genome materials or genomic information in South Korea. The Korean National Institute of Health (KNIH) is the primary national research agency in Korea, and is also responsible for genomic databases and biobanks (Table 1). The KNIH initiated the Korea Biobank Project (KBP) in 2008, including the establishment of the National Biobank of Korea (NBK) with 17 regional biobanks [27]. Through the KBP, the NBK has collected human biospecimens, consolidating them with donor clinical records, and shares them with biomedical researchers [28].

There are a number of regulations relating to human genome research and biobanks (Table 2). The most relevant legislation is the *Bioethics and Biosafety Act* (BBA), which was enacted in 2005 and fully revised in 2013. This act is generally applicable to all human subject research, genomic research, genetic testing, biobanks, stem cell research and embryonic research. In 2011, the *Guidelines for Protecting Personal Information in Medical Institutions* were approved by the Ministry of Public Administration and Security (MoPAS) and MoHW. Subsequently in 2013, the *Guidelines for Protecting Genomic Information* were developed by the Center for ELSI Research as a part of the National Project for Personalized Genomic Medicine 21 (PGM21) [32], in South Korea.

The BBA categorizes human samples (such as tissues, cells, blood and body fluids) and their components (such as serum, plasma, chromosomes, DNA and RNA) as human derivatives and sets regulations on human derivatives research. This categorization reflects the differences between genetics-related research and human subject research. The BBA has specified the establishment, role and organization of the National Bioethics Committee and the IRBs. The National Bioethics Committee is the highest level expert body designated by the President of South Korea. IRBs should be established in hospitals and research institutions that are involved in human subject research, stem cell and biospecimen research, genetic testing and biobanks. IRBs review the ethical and scientific validity of research protocols. Review for genomic research can be applied either through a fast or regular track, depending on whether human participants are involved. In 2002, IRB members founded the Korean Association of Institutional Review Boards (KAIRB) under the auspices of the Korean Academy of Medical Sciences [52]. By law, informed consent is generally required for the donation of human materials. The BBA Enforcement Rule provides the guidelines for official informed consent forms for donations as well as genetic tests.

As a recent public survey demonstrated, the development of professional genetic counseling is urgently required to improve the general population’s understanding of genomic medicine. After revision of the BBA, individual hospitals and research institutions are required to decide whether they will discard preserved biospecimens or transfer them to NBK after a fixed period. However, there are growing demands to expand the opportunities of researchers to access and distribute the qualified biospecimens with their associated data in NBK. In addition, direct-to-consumer genetic testing services now abound and they illustrate many loopholes in the BBA, which otherwise provides a very strict list of permissible genetic tests.

### Taiwan

Major research institutes conducting genome research in Taiwan include the Genomics Research Center (GRC) of the Academia Sinica, the National Health Research Institute (NHRI) and five National Centers of Excellence for Clinical Trials and Research established in university hospitals and sponsored by the Ministry of Health and Welfare (MOHW) (Table 1).

The *Human Biobank Management Act* (HBMA) was established in 2010 to regulate biomedical and genetic research and to ensure protection of human subjects. HBMA set out very detailed processes that must be observed in taking informed consent [33]. The *Human Subjects Research Act* (HSRA, 2011) even requires the Research Ethics Committee (REC) or IRB to be accredited by the MOHW first, before it can start reviewing research protocols. The National Accreditation Program for RECs and IRBs was started in 2004, and it is now compulsory for the institutional REC or IRB to receive regular visits and accreditation from the MOHW. Many RECs or IRBs in Taiwan have joined the FERCAP network, and 23 have been recognized by its SIDCER program. In comparison to other Asian countries, Taiwan has passed many legal regulations and policies to govern human and biobank research in recent years, which have raised potential conflicts between advancing biomedical or genomic research and protecting human subjects. Nonetheless, ELSI scholars of the National Research Program for Biopharmaceuticals (NRPB) have worked on mitigating such unnecessary conflict by improving clarity in legal and policy requirements, and by facilitating public consultation and engagement. The HBMA and HSRA restrict broad consent because these are not considered to provide enough information for the research subjects to consider and consent to. Consent is mostly directed at specific biomedical research and broad consent is allowed only in exceptional cases and after scientific and ethical reviews. Consent for the use of existing tissues is a major challenge in Taiwan. Consequently, RECs or IRBs play important roles in deciding the scope, feasibility and authorization of consents for leftover samples and in determining whether re-consent is required on a case-by-case basis.

The Taiwan Clinical Trials Consortium (TCTC), involving a dozen research institutions, has developed research collaborations and approaches for data sharing for major diseases. The Taiwan Biobank was launched in 2005 as part of Taiwan’s strategy to promote biomedicine and technology [29]. However, this project has been repeatedly criticized by specific human rights groups and legal scholars who have expressed concerns about genetic privacy, informed consent, linkage of databases, conflict of interest, procedural justice, and legitimacy of technology policymaking [53]. This has resulted in rigorous legislative and regulatory requirements as reflected in the HBMA and HRSA, which have limited the development of similar tissue storage or biobanking approaches and the access and utilization to such samples for the last few years.

Other ELSI issues of current interest in Taiwan include benefit sharing, return of and access to research results, workplace genetic discrimination, indigenous population research, commercialization of genetic testing, and issues related to big data research for healthcare.

## Comparing ELSI practices in East Asia

Here, we compare the ELSI practices and policies for human genomic research in the six East Asian countries, discussing research infrastructure, regulatory frameworks, ethics review and informed consent. We consider the local concerns and national interests, and the implications for wider research collaboration and public participation.

### Research infrastructure

Many of the large-scale genomic research initiatives in East Asia considered here are driven by interests defined by the state. This may indicate that researchers in these countries are less empowered to pursue research interests independently, when compared with their colleagues in Western countries. However, the globalization of genomic research could enable East Asian researchers to bypass their local academic societies and possibly national commitments [54] in favor of participation in international efforts like the Human Genome Organization (HUGO) [55] and the ICGC, and in Pan-Asian genomics initiatives like the HUGO Pan-Asian Population Genomics Initiative (PAPGI) [56], HUGO Pan-Asian SNP Consortium (PASNP) [20], the Asia Cohort Consortium [57], and Asia Pacific Society of Human Genetics (APSHG) [58]. Differing national interests have also resulted in different research infrastructures and establishments (Table 1). Governments in Japan and Taiwan have promoted comparable large-scale genotyping initiatives over the past decade, but the aims are different. Japanese initiatives are aimed at the making of science by and for the Japanese, whereas Taiwan’s initiatives convey a less pronounced message of nationalism [59].

### Regulatory frameworks

While many of the East Asian countries regulate informed consent, biobanking, sample sharing, material transfer, and ethics review of research (Tables 1 and 2), the regulations are not any more or less onerous than in Western countries. In general, regulatory control of biomedical research in East Asia has been indirect, encompassing guidelines that may not have any legal support. There is also wide variation in the manner and extent to which civil liberties are safeguarded. In Europe, the *Oviedo Convention on Human Rights and Biomedicine* has been very influential in promoting legislative initiatives against genetic discrimination in most of the European countries [60], but only a few East Asian countries have similarly targeted safeguards. In South Korea for instance, genetic discrimination is prohibited by legislation. However, there are built-in flexibilities that allow the use of genetic test results for future scientific developments, and it is in this regard less restrictive than the Oviedo Convention [61]. In Indonesia, safeguards against discrimination are directed at race and ethnicity rather than genetics *per se*, but they have been criticized as narrow by human rights groups [62]. These cases illustrate that political and cultural sensitivities or concerns, rather than research goals and initiatives, have shaped the scope of legislation on anti-discrimination. There are exceptions, where legislation has been targeted at specific research areas or practices regarded to be of particular concern or of national interest. For instance, many Asian countries have legislative safeguards for the use of personally identifiable information in research. These could, to some degree, be attributed to political pressure from Europe, and primarily the Data Protection Directive of the European Union [63]. In Indonesia, concerns over biopiracy have led to the enactment of the *Health Law* and the pronouncement of Ministry of Health regulations that give emphasis to safeguarding national sovereignty, especially in relation to biological resources and benefit sharing at a country level. These differences in ELSI practices are again more likely a consequence of socio-political concerns that are country-specific, rather than local application of universal ethical requirements [64].

### Ethics review and informed consent

In the United States of America (USA), IRBs review research proposals to ensure that they adhere to federal regulations for federally funded research. Modeled after this system, the establishment of IRBs or RECs for genomic research is based on regulations and guidelines in China, Indonesia, Japan and Singapore and on laws in South Korea and Taiwan. However, the application of these regulations and guidelines vary considerably in different institutions, as in the USA [65]. Common issues with regard to IRBs or RECs include the education and qualification of its members and the appropriate supervision of their work. In order to raise the quality of review, IRBs or RECs in Indonesia and Taiwan are under the supervision of government authorities and some have even joined international networks or are accredited under internationally recognized standards. Other East Asian countries have introduced annual evaluations (China), facilitated knowledge exchange with other board members (Japan, South Korea), and published detailed guidelines on the constitution, accreditation and operation of the boards (Singapore). Similar to the USA, differing standards have contributed to conflicts between IRBs or RECs and researchers [65-67].

Although gaining informed consent at an individual level is increasingly being emphasized in many East Asian countries, it continues to be a collective process in practice, where consent would also involve the research participant’s family members or members of his or her broader community [68]. As outlined above, this is particularly so in countries with a sizeable number of different ethnic minorities, such as China and Indonesia. However, the trend is that emphasis on individual decision-making at an individual level will continue to grow, and the conventional view of East Asian countries as being more family- or community-centered and less focused on individuals relative to Western societies may not be sustainable in the long run [69]. Regarding leftover tissue samples, there is also growing acceptance of general consent for their use in research, provided that appropriate safeguards are in place. Therefore, when comparing ELSI practices in East Asian and Western countries, a more accurate and up-to-date view may be that ethical, legal and social concerns that arise from genetic or genomic research are often shared, although differences arise in emphasis and approach. Another issue is the difficulty of ensuring that sample donors have acted voluntarily and on an informed basis. In some of the local communities and hospitals, resource-poor donors who may also be illiterate or uneducated are at risk of exploitation, particularly if refusal to contribute a sample is understood as denial of access to treatment.

Whether in East Asian or Western countries, a common lesson is that ethics review and informed consent need to be customized to the local contexts. Where research and policy experts in East Asia have been quick to embrace the Western and ‘international’ ELSI concepts and practices, there is now growing tension between these concepts and practices and those that arise from indigenous values. Arguably, the alleviation or resolution of such tensions necessitates the involvement of a broader range of interested stakeholders.

### Implications for research collaboration and public participation

In a number of East Asian countries, a combination of national interests and the hope of therapeutic benefit have been relied upon to garner public participation as well as support for particular, often large-scale, research initiatives. However, the failure to complement these initiatives with substantive public engagement might have led to public distrust in China, South Korea, Taiwan and Japan. In South Korea, national biobanks require that researchers be permitted to access and distribute the qualified biospecimens with their associated data. In Taiwan, the monopoly of authority (currently comprising a body of academic and technical experts) over the Taiwan Biobank has fostered tensions and widespread distrust, aggravated by a lack of open communication [70,71]. A similar lack of public trust is evident in Japan. Surveys showed that while a majority of the public approves of the promotion of genomic studies [72], public trust in science was damaged by events following the earthquake, tsunami and subsequent nuclear accident in 2011 [73]. In Singapore, the ethical evaluation of research initiatives is similarly expert driven, although it has a more consultative character as the national bioethics body has sought feedback from the relevant stakeholders and/or the general public on all of its deliberations and recommendations to the government. Some have observed that public discourses around science policies exist in Singapore but are limited by a lack of plurality and diversity of the participant communities [74].

## Conclusions and future directions

Many of the large-scale genomic research initiatives in East Asia are driven by national interests, and so differences in ELSI practices between countries are more likely to be a consequence of country-specific concerns. It is not unusual to find elements of Western or international governance approaches operating alongside more parochial ELSI practices in East Asian countries. Hence, regulatory control over biomedical research in these countries has a relatively mixed character, with direct oversight of some aspects and little or no control over others. In contrast, regulatory environments in Western countries (with the possible exception of the USA) have a more consistent legal character. Individual consent is increasingly highlighted and strictly followed as a matter of practice in East Asia, whereas some Western countries are attempting to relax informed consent as a strict requirement to facilitate scientific progress [75,76]. The absence of consistent ethical standards between the international research environment and national ones (as well as among local research institutions in some countries) could lead to conflicts and in the long run reduce public trust in East Asia.

To address these problems, East Asian countries should facilitate wider collaboration and public participation as well as appropriate training and supervision of IRBs or RECs to work towards harmonizing ELSI standards and practices. Where appropriate, more needs to be done to empower research participants to be involved as long-term interactive partners, as has been initiated in Western countries [77]. Ultimately, further understanding of the interactive dynamics between the global research agenda and shared local concerns will be needed to facilitate the wider involvement of East Asian countries in international genomic research. In addition, there is a need to promote public trust in research more generally through the consistent application of ethical and regulatory requirements, public engagement and cross-border collaborations. Further efforts will also be needed to understand ELSI practices and regulations in other countries in these and other global regions to promote international collaboration in human genomic research.

## Abbreviations

A*STAR: Agency for Science, Technology and Research, Singapore; APSHG: Asia-Pacific Society of Human Genetics; BAC: Bioethics Advisory Committee, Singapore; BBA: Bioethics and Biosafety Act, South Korea; BGI: Beijing Genomics Institute; CGM: RIKEN Center for Genomic Medicine; DDBJ: DNA Data Bank of Japan; EHR: Electric health record; ELSI: Ethical, legal and social implications; ERC: Ethics review committee; FERCAP: Forum for Ethical Review Committees in the Asian and Western Pacific Region; GRC: Genomics Research Center of the Academia Sinica, Taiwan; HBMA: Human Biobank Management Act, Taiwan; HGP: Human Genome Project; HSRA: Human Subjects Research Act, Taiwan; HUGO: Human Genome Organization; ICGC: International Cancer Genome Consortium; IRB: Institutional review board; JGA: Japanese Genotype-phenotype Archive; KAIRB: Korean Association of Institutional Review Boards; KBP: Korea Biobank Project; KCDC: Korean Centers for Disease Control and Prevention; KNEPK: National Research Ethics Committee, Indonesia; KNIH: Korean National Institute of Health; KSCDC: Kadoorie Study of Chronic Disease in China; METI: Ministry of Economy, Trade and Industry, Japan; MEXT: Ministry of Education, Culture, Sports, Science and Technology, Japan; MHLW: Ministry of Health, Labour and Welfare, Japan; MOH: Ministry of Health, China; MoHW: Ministry of Health and Welfare, South Korea; MOHW: Ministry of Health and Welfare, Taiwan; MoPAS: Ministry of Public Administration and Security, South Korea; MOST: Ministry of Science and Technology, China; NBDC: National Bioscience Database Center, Japan; NBK: National Biobank of Korea; NCBN: National Center Biobank Network, Japan; NHRI: National Health Research Institute, Taiwan; NRPB: National Research Program for Biopharmaceuticals, Taiwan; P3G: Public Population Project in Genomics and Society; PAPGI: Pan Asian Population Genomics Initiative; PASNP: HUGO Pan-Asian SNP Consortium; PGM21: Personalized Genome Medicine 21, South Korea; REC: Research ethics committee; SIDCER: Strategic Initiative for Developing Capacity in Ethical Review; TCTC: Taiwan Clinical Trials Consortium; TDR: Special Programme for Research and Training in Tropical Diseases; ToMMo: Tohoku Medical Megabank Organization, Japan; WHO: World Health Organization.

## Competing interests

The authors declare that they have no competing interests.

## Authors’ contributions

WZ and CH drafted the subsection on China. YS drafted the subsection on Indonesia. IL and HK drafted the subsection on South Korea. DFCT drafted the subsection on Taiwan. CW-LH drafted the subsection on Singapore, participated in cross-country comparisons and edited the whole manuscript. KK drafted the subsection on Japan and participated in cross-country comparisons. JM designed the approach for cross-country comparisons and edited the subsection on Japan. GY drafted the subsection on Japan, conducted the cross-country comparisons and edited the whole manuscript. All authors read and approved the final manuscript.
